# Thirty-two years post-Chernobyl: risk perception about radiation and health effects among the young generation in Gomel, Republic of Belarus

**DOI:** 10.1093/jrr/rry079

**Published:** 2018-09-22

**Authors:** Rei Ohkuma, Jumpei Takahashi, Tamara Sharshakova, Anastasiya Sachkouskaya, Anatoly Lyzikov, Evgenii Voropaev, Dmitrii Ruzanov, Makiko Orita, Yasuyuki Taira, Noboru Takamura

**Affiliations:** 1 Department of Global Health, Medicine and Welfare, Atomic Bomb Disease Institute, Nagasaki University, Nagasaki, Japan; 2 Gomel State Medical University, Gomel, The Republic of Belarus

*To the editor*:

Thirty-two years have passed since the 1986 accident at the Chernobyl Nuclear Power Plant (CNPP). It is well known that thyroid cancer has dramatically increased among children and adolescents who were exposed to radioactive iodine released at the accident site in Belarus, Russia, and Ukraine [1, 2]. In 2016, the World Health Organization (WHO) updated the assessment of health effects of the accident and reported that according to national studies in the three countries, more than 11 000 cases of thyroid cancer had been diagnosed by 2016 [3]. Apart from thyroid cancer, there is no scientific evidence for radiation-associated increase in any health effects, including genetic effects in residents around the CNPP. The WHO has emphasized the importance of mental health problems in these residents and the necessity of providing mental health support for these residents.

After the Fukushima Daiichi Nuclear Power Station accident, the risk perception of the residents there was evaluated, and many residents were found to have anxiety about the health effects of children and genetic effects in the next generation in Fukushima [4, 5]. Risk perception among the young residents in the areas around Chernobyl has not yet been fully investigated. Gomel in the Republic of Belarus was severely contaminated by the accident, and the incidence of thyroid cancer among residents of this region was dramatically increased. Therefore, in the present study we investigated the risk perception of young residents in the Gomel region, Republic of Belarus, who were born after the CNNP accident.

We initially invited 200 students in the 3rd and 4th years of Gomel Medical University (Republic of Belarus) who were born in Gomel. After obtaining informed consents, we included 189 students in this study and distributed questionnaires among the students. The questionnaire contained questions related to sociological factors and risk perception regarding radiation exposure and related health effects. A total of 56 students (29.6%) responded that they experienced ‘anxiety that they were born in the Gomel region’ and 44 students (23.3%) said they experienced ‘anxiety to consume locally produced foods in Gomel’. Furthermore, 102 students (54.0%) said they experienced ‘anxiety about health effects due to radiation exposure by living in present-day Gomel region’, and 104 students (55.0%) had ‘anxieties about the genetic effects that might be passed on to the next generation as a result of living in present-day Gomel region.’ Logistic regression analysis revealed that ‘anxiety about health effects due to radiation exposure by living in present-day Gomel region’ was independently associated with ‘anxiety that they were born in Gomel region,’ and ‘anxieties about the genetic effects that might be passed on to the next generation as a result of living in present-day Gomel region’ (Table 1).

**Table 1.** Logistic regression analysis for ‘anxiety about health effects due to radiation exposure from living in the present-day Gomel region’ among young residents of Gomel, Republic of Belarus

Variables^a^	Units	OR	95% CI	*P* value
**Gender**	Male/Female	0.99	0.42−2.35	0.99
**Anxiety that they were born in the Gomel region**	Yes/no	5.65	2.42–13.17	<0.001
**Anxieties about the genetic effects that may be passed on to the next generation as a result of living in the present-day Gomel region**	Yes/no	7.90	3.94–15.87	<0.001

OR = odds ratio, CI = confidence interval.

^a^We did not make further adjustment for other covariates.

Although the number of the study participants was relatively limited, our preliminary results showed that even 32 years after the incident, young residents of the area around the CNPP continue to have anxiety about health and genetic effects. None of the study participants were ever exposed to radioiodine from the CNPP because they were all born after the accident. Nevertheless, more than half of the students had anxiety regarding the potential health effects caused by radiation exposure and the genetic effects that may be passed on to the next generation. Thus, education and risk communication in the field of radiation health science is vital [6].

Furthermore, these findings strongly suggest the importance of education in the field of radiation health science in Japan. To overcome the societal prejudices toward Fukushima based on incorrect information, we need to establish an education system in schools to spread awareness in the field of radiation health science.
